# Supplementary material for: Projected rapid response of stratospheric temperature to stringent climate mitigation
Source: Nat Commun. 2024 Aug 3;15:6590. doi: 10.1038/s41467-024-50648-8 (PMC11297936; doi:10.1038/s41467-024-50648-8)
Supplement: Supplementary file 1 — Supplementary Information [file 41467_2024_50648_MOESM1_ESM.pdf]

Projected rapid response of stratospheric temperature to stringent climate mitigation

Supplementary Information

Grasiele Romanzini-Bezerra[1] and Amanda C. Maycock[1*]

[1]School of Earth and Environment, University of Leeds, UK

[*]Corresponding author: a.c.maycock@leeds.ac.uk

Content:

1. Supplementary Figures 1 and 2

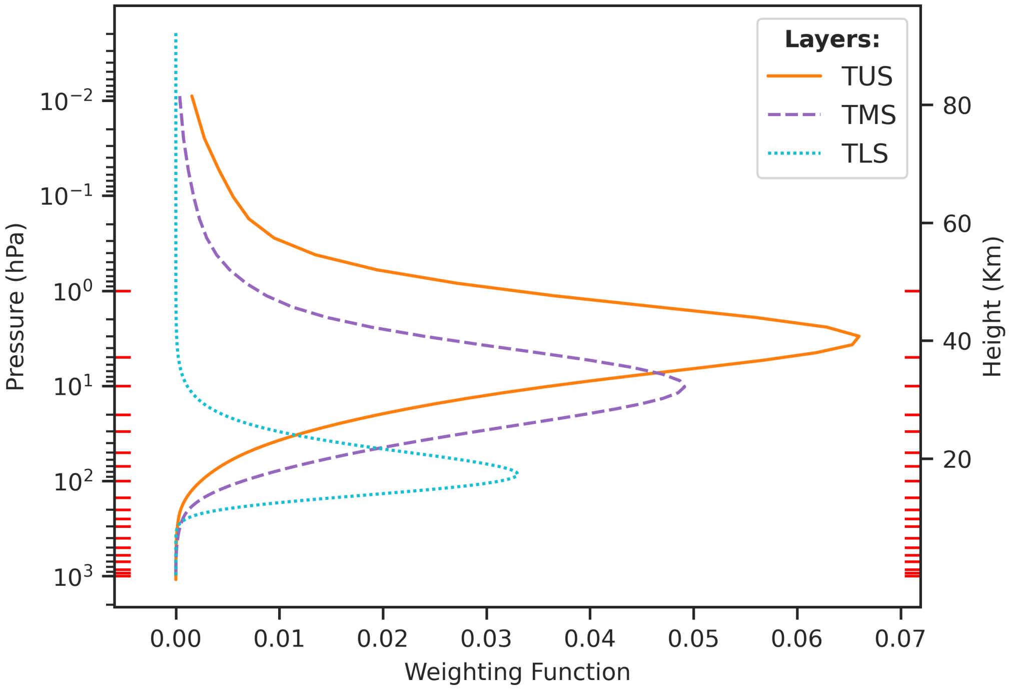

Supplementary Figure 1: Atmospheric weighting functions applied to climate model data. Weighting functions correspond to temperature of the: lower stratosphere (TLS), middle stratosphere (TMS) and upper stratosphere (TUS). Red ticks show the location of the model pressure levels.

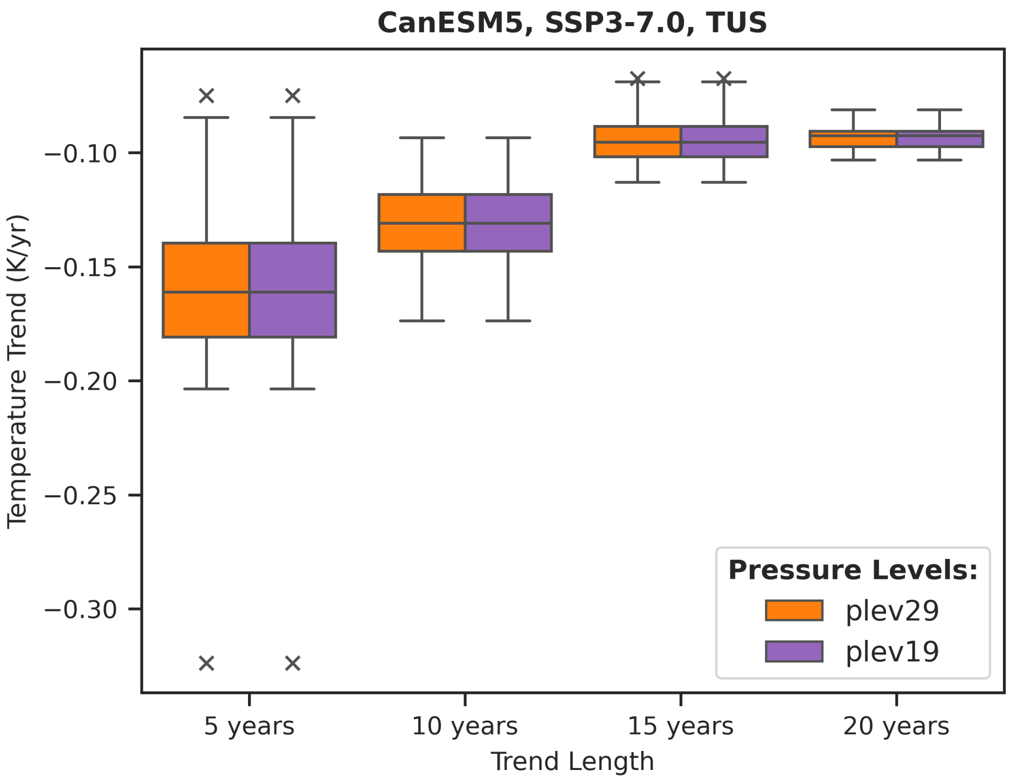

Supplementary Figure 2: Dependence of TUS trends on temperatures above the stratopause. Recalculation of near-term TUS trends for the SSP3-7.0 scenario in CanESM5 using the CMIP6 'plev19' variable from the main paper and the 'plev29' variable, which includes 10 additional layers at pressures less than 1 hPa. Boxes denote 25-75th percentile range, whiskers denote 10-90th percentile range and crosses denote outliers.
